# Imaging analysis of Parkinson’s disease patients using SPECT and tractography

**DOI:** 10.1038/srep38070

**Published:** 2016-11-30

**Authors:** Seong-Jin Son, Mansu Kim, Hyunjin Park

**Affiliations:** 1 Department of Electronic, Electrical, and Computer Engineering, Sungkyunkwan University, Suwon, Korea; 2 School of Electronic and Electrical Engineering, Sungkyunkwan University, Korea; 3 Center for Neuroscience Imaging Research (CNIR), Institute for Basic Science, Korea

## Abstract

Parkinson’s disease (PD) is a degenerative disorder that affects the central nervous system. PD-related alterations in structural and functional neuroimaging have not been fully explored. This study explored multi-modal PD neuroimaging and its application for predicting clinical scores on the Movement Disorder Society-sponsored Unified Parkinson’s Disease Rating Scale (MDS-UPDRS). Multi-modal imaging that combined ^123^I-Ioflupane single-photon emission computed tomography (SPECT) and diffusion tensor imaging (DTI) were adopted to incorporate complementary brain imaging information. SPECT and DTI images of normal controls (NC; n = 45) and PD patients (n = 45) were obtained from a database. The specific binding ratio (SBR) was calculated from SPECT. Tractography was performed using DTI. Group-wise differences between NC and PD patients were quantified using SBR of SPECT and structural connectivity of DTI for regions of interest (ROIs) related to PD. MDS-UPDRS scores were predicted using multi-modal imaging features in a partial least-squares regression framework. Three regions and four connections within the cortico-basal ganglia thalamocortical circuit were identified using SBR and DTI, respectively. Predicted MDS-UPDRS scores using identified regions and connections and actual MDS-UPDRS scores showed a meaningful correlation (*r* = 0.6854, *p* < 0.001). Our study provided insight on regions and connections that are instrumental in PD.

Parkinson’s disease (PD) is a degenerative disorder of the central nervous system that mainly affects the motor system^1^. PD-related deaths were expected to increase by 28.2% from 43.7 thousand deaths in 1990 to 102.5 thousand deaths in 2013^2^. In most patients, PD is idiopathic with little evidence of pathophysiology^3^. The most obvious symptoms are movement-related, such as rigidity, shaking, and slowness of movement^4^. These symptoms are associated with a loss of dopaminergic neurons in the substantia nigra (SN) and those that project to the striatum^5^,^6^. This dopamine imbalance causes inhibition of basal ganglia output and dysfunction within the cortico-basal ganglia thalamocortical (CBGT) circuit that consists of the associative cortex, limbic cortex, sensorimotor cortex, caudate, putamen, thalamus, and pallidum^5^,^6^.

Single-photon emission tomography (SPECT) is commonly used for PD diagnosis^7^. SPECT imaging using ^123^I-Ioflupane (^123^I-Ioflupane-SPECT) provides information based on local binding of presynaptic dopamine transporters (DaTs) with ^123^I-Ioflupane, which has been shown to be highly correlated with PD progression^7^,^8^. This binding measure is quantitative and assesses the spatial distribution of dopamine transporters. Furthermore, ^123^I-Ioflupane-SPECT is an imaging modality that is capable of differentiating between PD and essential tremor^9^. SPECT imaging can also distinguish between PD and drug-induced Parkinsonism^9^,^10^. However, any disease that causes loss of the presynaptic dopamine neurons will appear as abnormal compared with normal controls (NCs)^11^. Thus, SPECT is not able to differentiate among PD, progressive supranuclear palsy, multiple system atrophy, and other neurodegenerative disorders that affect the dopamine neurons^11^. Most studies that use ^123^I-Ioflupane-SPECT have focused on the striatum (i.e., putamen and caudate)^12^,^13^,^14^,^15^. Researchers have reported that PD has markedly reduced dopamine transporter levels in the striatum, which are correlated with disease progression and clinical scores^12^,^13^,^14^,^15^. Only a few studies have investigated the CBGT circuit using SPECT imaging^16^,^17^. The specific binding ratio (SBR) is the ratio of the concentrations of the specific binding radioactivity of a target brain region to the non-specific binding radioactivity of the reference region that is devoid of DaT uptake. The occipital cortex and cerebellum are commonly adopted reference regions that have similar performances^18^. We chose the occipital region as the reference region, which was similar to existing studies^18^,^19^.

Diffusion tensor imaging (DTI) is a widely-used MRI imaging technique that can be used to diagnose various neurodegenerative diseases^20^,^21^,^22^,^23^. DTI can provide information about *in vivo* neuronal fibers using anisotropic water diffusion in white matter^8^. DTI data are processed with an algorithm known as tractography in order to extract relevant fiber information^24^. The processed fiber information is assessed with a connectivity analysis that allows observation of the whole brain as a complex connected network^25^. One DTI study reported structural connectivity deficits in sensorimotor circuitry within the CBGT circuit of PD^26^. Functional connectivity deficits were also found in the same circuitry using resting-state functional MRI (rs-fMRI)^26^.

In this study, we explored multi-modal neuroimaging, which uses both SBR of SPECT and track density of DTI in the CBGT circuit to better characterize PD and explain PD-related clinical scores.

## Results

### Significant differences in regions using SBR

**Figure 1:**
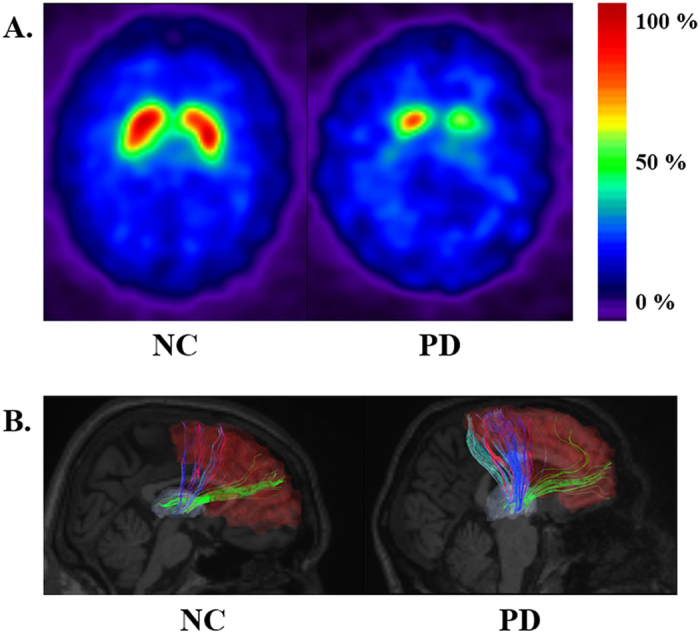
Representative SPECT images and fiber tracts obtained from DTI of NC and PD cases. **(A)** The SPECT images were representative slices taken from registered 3D volumes. Note the difference in putamen and caudate regions. **(B)** Fiber tracts obtained from DTI tractography focusing on associate cortex – thalamus connection. Associative cortex was colored in red and thalamus was colored in light purple. Note the increased number of fiber connecting the regions in the PD case compared to NC case.

Representative SPECT images for PD and NC groups were given in Fig. 1. SPECT images were shown as SBR was computed from SPECT. The images focused on putamen and thalamus. Group-wise differences in SBR between NC and PD subjects were quantified using permutation tests (see the Methods section). The associative cortex, putamen, and globus pallidus are among the seven regions within the CBGT circuit, and they showed significant (p < 0.05, corrected) differences between PD and NC subjects in SPECT analysis. The putamen and globus pallidus showed the largest difference (p < 0.001). The associative cortex (p < 0.003) was also significantly different between PD and NC subjects. These regions have been previously reported as important regions related to dopamine transmission^27^,^28^. The detailed results are shown in Table 1.

### Significant differences in connections using DTI

Representative image of fiber tracts obtained from DTI tractography for PD and NC groups were given in Fig. 1. The images focused on associate cortex – thalamus connection. Group-wise differences in fiber density between NC and PD subjects were also quantified using permutation tests (see the Methods section). The associative cortex – thalamus, limbic cortex – caudate, limbic cortex – putamen, limbic cortex – thalamus, globus pallidus – putamen, and globus pallidus – thalamus connections among the 13 connections within the CBGT circuit showed significant (p < 0.05, corrected) differences between NC and PD subjects in DTI analysis. Furthermore, four identified connections contained previously identified regions using SBR, which included the associative cortex, putamen, and globus pallidus. The detailed results are shown in Table 2.

### Predicting MDS-UPDRS scores

**Figure 2:**
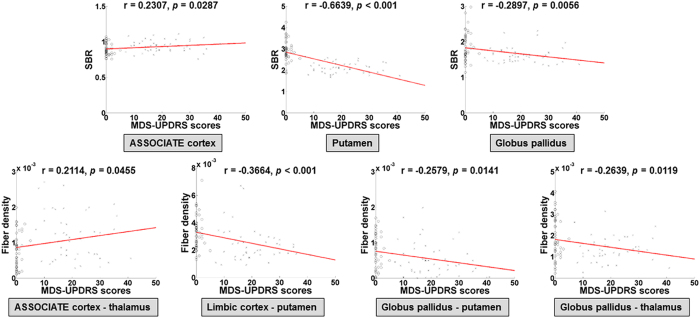
Correlation between imaging features (SBR and fiber density) and MDS-UPDRS scores. SBR of identified ROIs were correlated with MDS-UPDRS scores (first row). Fiber density of identified connections were correlated with MDS-UPDRS scores (second row).

Pearson correlations between the chosen imaging features (i.e., SBR value of ROIs and fiber density of connections) and Movement Disorder Society-sponsored Unified Parkinson’s Disease Rating Scale (MDS-UPDRS) scores were plotted in Fig. 2. All chosen SBR values of ROIs and fiber density of connections showed significant correlation (p < 0.05) with MDS-UPDRS. The SBR of associative cortex and fiber density of associative cortex – thalamus connection were negatively correlated with MDS-UPDRS scores. Other SBR values of chosen ROIs and fiber density of chosen connections showed positive correlation with MDS-UPDRS. Among the identified regions and connections, putamen showed the highest correlation with MDS-UPDRS.

Partial least squared regression (PLSR) was performed to identify possible links between identified regions and connections and MDS-UPDRS, as shown in Tables 1 and 2. Identified imaging features were used as independent variables, and MDS-UPDRS scores were used as the dependent variable. The PLSR results of three identified regions are shown below. The SBR of the associative cortex, putamen, and globus pallidus regions explained the MDS-UPDRS scores (explained variance = 0.3831). Among the six connections that were found to have significant differences between NC and PD, we retained the connections that were previously identified in SPECT analysis. The PLSR results of four identified structural connections are shown below. The fiber density of the associative cortex – thalamus, limbic cortex - putamen, globus pallidus – putamen and globus pallidus – thalamus connections provided explanations for the MDS-UPDRS score (explained variance = 0.2888). The degree of explanation was higher when the SBR values, rather than the DTI connections, were used. Furthermore, all SBR values of the three regions and the fiber density values of four connections were used as independent variables. These results provided the best explanation of the MDS-UPDRS scores (explained variance = 0.5244).

**Figure 3:**
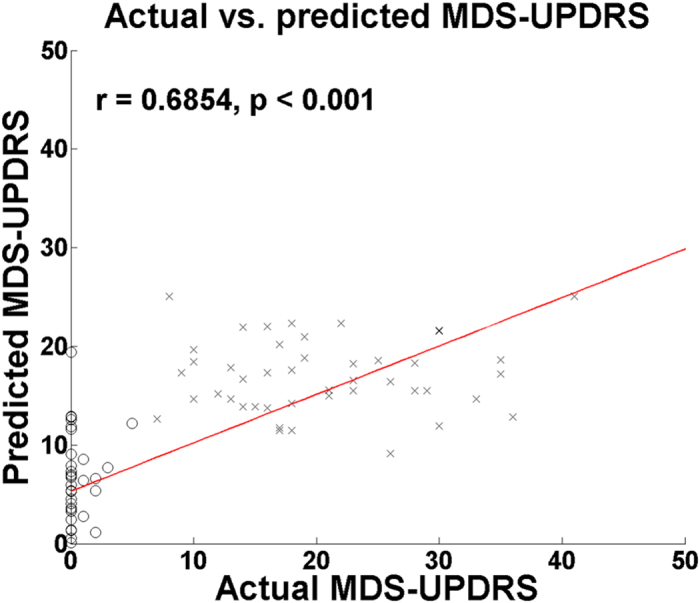
Comparison of actual and predicted MDS-UPDRS between PD and NC subjects. There was significant correlation (*r* = 0.6854, *p* < 0.001) between predicted MDS-UPDRS scores and actual MDS-UPDRS scores using SBR of identified regions (associative cortex, putamen, and globus pallidus) and fiber density of connections (associative cortex – thalamus, limbic cortex – putamen, globus pallidus – putamen, and globus pallidus – thalamus).

Leave-one-out cross validation (LOOCV) was performed to validate the predicted MDS-UPDRS scores against the actual MDS-UPDRS scores. The number of latent variables (LVs) in the PLSR framework showed minimal change for different training sets within the leave-one-out cross validation. The most used number of LVs was one when using all identified regions and connections (from both SPECT and DTI), one when using only identified regions from SPECT, and three when using only identified connections from DTI. Similar to number of LVs, the regression coefficients (β) could change for each training set. The most frequently used regression coefficients for using both SPECT and DTI, only DTI, and only SPECT were reported in the supplement. There was a high correlation between predicted MDS-UPDRS scores and actual MDS-UPDRS scores using all identified regions and connections (*r* = 0.6854, *p* < 0.001) compared with using the identified regions from SPECT (*r* = 0.5843, *p* < 0.001) and the identified connections from DTI (*r* = 0.4665, *p* < 0.001; Fig. 3). The mean root mean squared (RMS) error between predicted and actual MDS-UPDRS was 8.46 with a standard deviation of 8.46 using both SPECT and DTI.

## Discussion

This study adopted a multi-modal approach combining ^123^I-Ioflupane-SPECT and DTI to incorporate complementary neuroimaging information for PD patients and to predict MDS-UPDRS scores using identified neuroimaging features. We improved performance by using multi-modal neuroimaging features compared to single-modal neuroimaging features in terms of explained variance. We found regions and structural connections that could distinguish between PD and NC. We believe those regions and connections could be used as a baseline for future PD research. PD is often misdiagnosed up to 35% because the characteristic of the PD is not fully understood^29^. Thus, we believe identifying neuroimaging biomarkers of PD and predicting progression of PD based on neuroimaging biomarkers is still worthwhile.

In the present study, we focused on the CBGT circuit among many brain circuits. PD results from a loss of dopaminergic neurons in the pars compacta of the SN and a resulting reduction in the level of dopamine in the striatum^30^. The loss of dopamine is related to profound changes in neuronal activity in the CBGT circuit^5^. The CBGT consists of the striatum, globus pallidus, and thalamus^6^. These structures are part of larger functional and anatomical parallel circuits that also include areas of the frontal cortex and of the ventral thalamus. Depending on the function of the frontal cortex, these CBGT circuits are designated as limbic, associative, and sensorimotor cortices^5^,^6^.

We found the associative cortex, globus pallidus, and putamen regions to be significant in explaining the difference between PD and NC. The frontal lobe or prefrontal association complex within the associative cortex is involved in planning actions and movement, as well as abstract thought^31^. The dorsolateral prefrontal, lateral orbitofrontal, parietal and temporal association areas regulate the function of the caudate and putamen, which is highly innervated by dopamine neurons that originate from the substantia nigra^32^. The globus pallidus has been reported to play important roles in the CBGT circuit. The sub-thalamic nucleus might become overactive and act as a brake on the globus pallidus, causing motion shutdown and rigidity in PD^33^,^34^. When the globus pallidus is overstimulated, it exerts an over-inhibitory effect on the thalamus, decreasing the thalamus output and causing tremor^34^. The DaT concentration is highest in the putamen and caudate where the DaTs are located on the presynaptic side of dopaminergic synapses^35^. The putamen plays a role in the selection of movement and the automatic performance of previously learned movements^36^. The putamen in the CBGT circuit also plays a key role because its inputs and outputs are interconnected to the globus pallidus^37^.

Clinical variables were predicted using PLSR, with SBR and fiber density as independent variables. The SBR of identified regions provided an explanation for the MDS-UPDRS scores with an explained variance of 0.3831. The fiber density of identified connections explained MDS-UPDRS scores with an explained variance of 0.2888. Using both SBR and fiber density values led to the best explained variance of 0.5244. We conjecture that SPECT and DTI provide complementary information for distinguishing between PD and NC, as shown by the correlations with MDS-UPDRS scores. Predicted MDS-UPDRS scores were also validated with actual MDS-UPDRS scores using LOOCV. The MDS-UPDRS scores were predicted using the PLSR, and comparison between actual and predicted MDS-UPDRS scores showed a high correlation (*r* = 0.6854, *p* < 0.001). We predicted MDS-UPDRS scores using imaging features that could distinguish between NC and PD groups well. Both NC and PD groups were used to generate the independent variables to predict MDS-UPDRS score. There were unusually high of number cases with MDS-UPDRS score zero coming from the NC group. We removed 34 NC cases from the first interval (0~9), which leads to having 14 cases (11 NC and 3 PD), to have evenly distributed number of cases covering all four intervals and re-performed the prediction analysis. The correlation between actual and predicted MDS-UPDRS scores was still significant (r = 0.5949, *p* < 0.001) compared to the results using all NC cases (*r* = 0.6854, *p* < 0.001). Details using less number of NC cases were provided in the supplement.

We aimed to investigate the CBGT circuit, which was divided into seven regions as was done in a previous study^26^. Voxel-based analysis would identify regions in the whole brain that are not necessarily from the CBGT circuit, which is outside of the scope of our study. Hence, we leave this approach as future research. ^123^I-Ioflupane SPECT has main application in the striatal regions but extrastrial regions could also be explored. Kaansinen *et al*. explored the effects of aging and gender on striatal and extrastriatal regions including striatum, thalamus, globus pallidus, frontal cortex, temporal cortex for NC and PD patients^38^. Frosini *et al*. also used ^123^I-Ioflupane SPECT to striatal and extrastriatal regions to assess the differences between PD patients with depression and without depression^39^. There were significant differences in left cingulate cortex between the comparison groups. Moreover, Eusebio *et al*. used ^123^I-Ioflupane SPECT to explore the effects of age and gender at the scale of a single SPECT voxel level for normal controls^40^. The SBR decreased with age not only in the striatal region, but also in extrastriatal regions, especially in anterior cingulate, medial frontal and insulo-opercular cortices. They also found increased SBR values in striatal and extrastriatal regions for women compared to men.

Globus pallidus is a small region and thus SBR of globus pallidus could be affected by spill-over from the nearby putamen. The average size of globus pallidus in one dimension is considered to be 1.5–3 times the spatial resolution of SPECT^41^,^42^. As globus pallidus was larger than the spatial resolution of SPECT, we believe spill-over from putamen will not significantly alter SBR statistics of globus pallidus. In addition, partial volume effect (PVE) correction was applied so that spill-over effect could be reduced.

One limitation of our study was that we did not consider longitudinal PD data. Imaging findings related to NC and PD might fluctuate from baseline to follow-up imaging sessions. Those longitudinal changes in imaging findings lead to the noise floor that are inherent in the imaging process. With an established noise floor, we can better assess group-wise differences. Unfortunately, such longitudinal data were missing from the Parkinson’s Progression Markers Initiative (PPMI) database. We leave this focus as an important topic for future research.

We identified three regions and four connections that distinguished between PD and NC patients using SBR for SPECT and fiber density for DTI. The identified regions and connections were also well-correlated with the MDS-UPDRS scores. These findings confirmed that important regions and connections are correlated with well-established clinical scores. Our study provided insight into the regions and connections involved in PD, and additional research is needed to better understand the roles of these connections in PD progression.

## Methods

### Subjects and imaging data

This study was a retrospective analysis of anonymized data, and institutional review board (IRB) approval was obtained at Sungkyunwkan University. Our study was performed in full accordance with the local IRB guidelines. Informed consent was obtained from all subjects. In this study, we obtained diffusion MRI and SPECT images from the research database of the PPMI. The subjects consisted of 90 participants who were classified as NC (n = 45) or PD (n = 45) in matched age and sex ratios. Only 45 NC subjects had both MRI (i.e., DTI, T1-, and T2-weighted imaging) and SPECT data available in the database, and we were limited to 45 subjects in the NC group. We randomly chose 45 patients with MRI and SPECT data in the PD group to have a matched number of cases. The following imaging data were available for all subjects: ^123^I-Ioflupane SPECT, diffusion MR, and T1- and T2-weighted MRI. The groups were classified according to the criteria set by the PPMI consortium^43^. Details on the subjects, including MDS-UPDRS scores, are listed in Table 3. All MRI images were obtained using a Siemens 3T scanner. The following protocol was used for SPECT image acquisition. ^123^I-Ioflupane (185 MBq) was injected in each patient at least 30 min after potassium perchlorate (400 mg) oral administration to prevent radiolabeled iodine thyroid uptake. Subjects underwent SPECT imaging within 4 h (±30 min) after the injection by a dual-head gamma camera (Millennium VG; General Electric Medical Systems, Milwaukee, WI, USA) equipped with low-energy high-resolution collimators. Raw projection data were acquired into a 128 × 128 matrix of 120 projections with two 20% symmetric photopeak windows centered on 159 KeV and 122 KeV. The images were reconstructed and attenuation was corrected, implementing either filtered back-projection or an iterative reconstruction algorithm using standardized approaches. After processing, images were spatially- and intensity-normalized into images with dimensions of 91 × 109 × 91 with 2 × 2 × 2 mm^3^ voxel resolution. Diffusion images were acquired with a Siemens 3 T scanner using the following parameters: b = 1000 s/mm^2^, 64 diffusion gradient directions with one b0 image, image matrix = 116 × 116 × 72, and voxel resolution = 1.98 × 1.98 × 2 mm^3^. The subjects also underwent T1- and T2-weighted MR imaging as well as the image pre-processing steps required them. Acquisition parameters for the T1-weighted images were as follows: TR = 2300 ms, TE = 2.98 ms, TI = 900 ms, image matrix = 240 × 256 × 176, and voxel resolution = 1 × 1 × 1 mm^3^. The parameters for the T2-weighted images were as follows: TR = 3,000 ms, TE = 101 ms, image matrix = 228 × 256 × 48, and voxel resolution = 0.9375 × 0.9375 × 3 mm^3^.

### Image pre-processing

**Figure 4:**
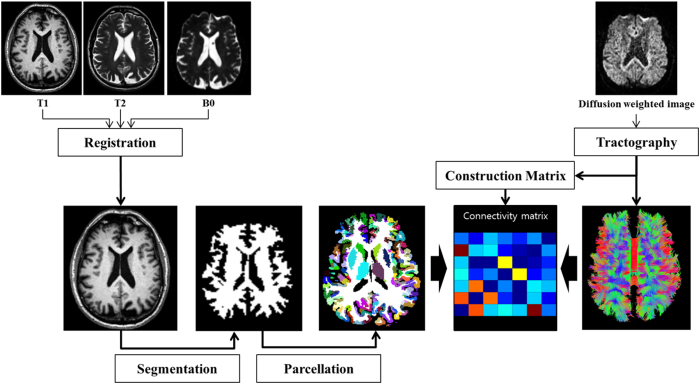
Overall image processing procedures of DTI. Image preprocessing was performed using co-registration among images to normalize onto the common space. ROI specification was performed by co-registration within the CBGT circuit. A tractography algorithm was applied to the preprocessed DTI images to compute fiber information. Fiber density was calculated using the outcomes of the processed DTI results.

Both MRI and SEPCT data require pre-processing. There are some excellent review articles on this procedure; thus, only a brief summary is given below^44^,^45^. The raw SPECT data were iteratively reconstructed using a hybrid-ordered subset expectation maximization (OSEM) algorithm in a HERMES workstation (Hermes Medical Solutions, Stockholm, Sweden)^44^. The reconstructed images underwent attenuation correction. The partial volume effect is known to occur in boundary voxels where a given voxel contains more than two tissue types. The PVE was corrected using the Müller-Gärtner’s method in the PVE lab pipeline program v2.2^49^. The uncorrected SPECT image and T1-weighted structural MR image were used as input to the PVC algorithm for voxel-wise partial volume effect correction^46^. The raw MRI data were pre-processed using the following steps. MRI pre-processing was performed using the Connectome Mapping Toolkit (CMTK) and Python-based open-source software (www.cmtk.org). For each subject, a T2-weighted image was used in an intermediate step to achieve image co-registration between the T1-weighted image and the diffusion MRI image. The T1-weighted image was first linearly registered onto the T2-weighted image. Then, the T2 weighted image was subsequently non-linearly registered onto the b0 image (i.e., part of diffusion MRI image). We than concatenated the spatial transforms obtained from the two image registration results to achieve co-registration between the T1-weighted and b0 images. This two-step process was less prone to the distortion that is present in the diffusion MRI than a direct registration between the T1 weighted and diffusion images^45^. The registered T1-weighted image was segmented into grey matter, white matter, and cerebrospinal fluid (CSF) using FreeSurfer. The segmented white matter was later used to guide the tractography algorithm. All pre-processing steps were performed using CMTK, FSL, FreeSurfer, MIAMI fuse and in-house MATLAB code^45^,^47^,^48^,^49^. The complete image pre-processing procedures of MRI are provided in Fig. 4.

### ROI specifications

**Figure 5:**
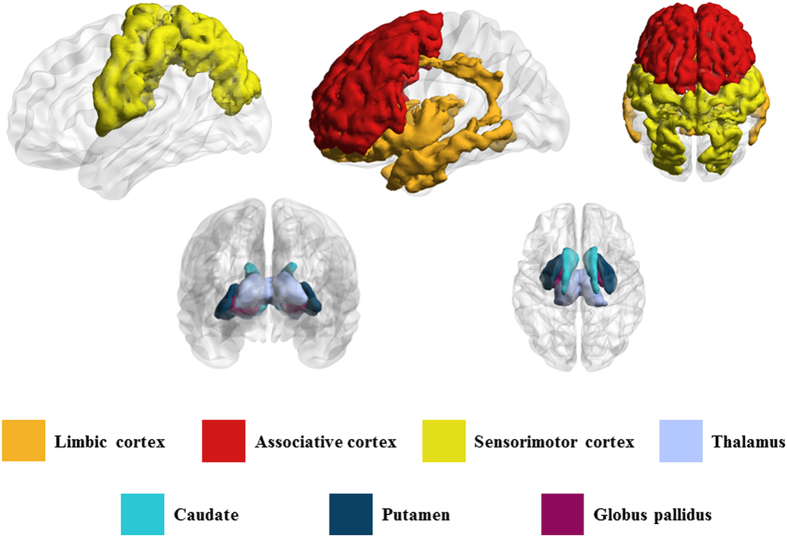
ROI specifications. A total of seven ROIs were defined as the limbic, associative, sensorimotor cortex, thalamus, caudate, putamen, and globus pallidus.

ROI specification is required for calculating SBR using SPECT images and for calculating correlations between specific ROIs for connectivity analysis using DTI data. Our analysis focused on the CBGT circuit, which was comprised of seven regions, caudate, putamen, pallidum, thalamus, sensorimotor cortex, associative cortex, and limbic cortex, as shown in Fig. 5. Three ROIs, limbic, associative, and sensorimotor cortex of the CBGT circuit, were larger than the remaining five ROIs. The larger ROIs could be further sub-divided into smaller regions, however, this would make the multiple comparison issue more severe, thus we avoided such sub-division. The sensorimotor cortex, which includes the pre-central, post-central, and para-central gyri (Brodmann areas 1–5), is related to motor symptoms in PD. The associative cortex, which includes the dorsolateral prefrontal, middle, and superior frontal cortices (Brodmann areas 8, 9, 44–47), is related to executive function and is affected by aging and PD. The limbic cortex, which includes the medial temporal, orbitofrontal, posterior, and anterior cingulate cortices, insula, entorhinal, hippocampus, and amygdala, are related to stuttering and other movement disorders. All seven regions are structurally connected to the striatum.

**Figure 6:**
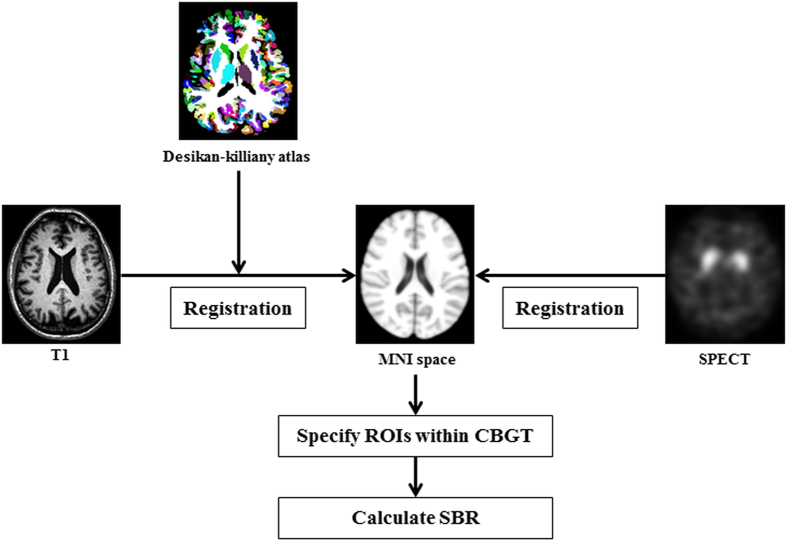
Overall image processing procedures of SPECT. The processing of SPECT image was similar to that of the DTI processing. The images were co-registered to common space (MNI space) to define the ROIs within the CBGT circuit. The mean intensity of each ROI was used to compute the regional SBR.

ROI specifications were achieved by image co-registration. Image co-registration mapped the atlas information onto the subject’s image space so that both images resided on the same coordinate space and so that pre-defined ROI information of an atlas could be applied to the subject’s image. Three cortical structures, sensorimotor, associative, and limbic cortex, and four subcortical structures, caudate, putamen, globus pallidus, and thalamus, were defined by the Desikan-Killiany anatomical atlas using spherical transform and Bayes rule cost function in Freesurfer^48^. ROI specifications for the SPECT data were as follows. The Desikan-Killiany atlas was transformed onto the SPECT image of each subject using a 9 DOF spatial transform and mutual information cost function in MIAMI fuse^49^. The ROI specification procedures for SPECT data are provided in Fig. 6. The combined atlas was applied to the DTI data, and the entire procedure for DTI data analysis are provided in Fig. 4.

### Computing the specific binding ratio

The ^123^I-Ioflupane SPECT radiotracer produces low-level, non-specific dopamine transporter bindings in addition to the intended specific dopamine transporter bindings^50^. In this study, SBR was computed for seven striatal and extra-striatal regions. The SBR is defined as the ratio between the concentrations of the specific binding radioactivity of target ROI to the non-specific binding radioactivity of the reference region^50^,^51^. The non-specific binding uptake is estimated from a brain region devoid of ^123^I-Ioflupane, which was chosen as the occipital region^18^. The occipital region was specified by image co-registration with the Desikan-Killiany anatomical atlas using procedures described in ROI specifications. SBR was calculated for seven regions (caudate, putamen, pallidum, thalamus, sensorimotor cortex, associative cortex, and limbic cortex) according to the following formula: SBR = (mean voxel level of a specific region)/(mean voxel level of the occipital region) - 1.

### Tractography and structural network construction

A tractography algorithm was applied to the DTI data to compute fiber information. Image distortions caused by eddy currents and head motions during DTI acquisition were corrected using FSL’s Eddy current tool and MCFLIRT^47^. Whole brain white matter fiber information was computed using the fiber assignment of the continuous tracking (FACT) algorithm included in the Diffusion Toolkit^52^. The FACT algorithm propagated a fiber tract from the center of a seed voxel along the direction of the dominant vector, which was determined by the largest eigenvector of the tensor until the tract exited to the next voxel. The intercept of the previous voxel was used as the starting point of the next voxel. Tracking was complete when the algorithm entered a region with an unexpected change in fiber direction (i.e., an angle threshold greater than 60°). Tractography was limited to white matter regions and their neighbors, as neuronal fibers mainly exist in the white matter. Every voxel was considered a seed voxel, and we kept only fibers whose end-points were within the pre-defined ROIs.

DIT-derived connectivity is commonly referred to as structural connectivity, while functional connectivity is derived from functional MRI. Structural connectivity was computed using seven regions as nodes and 13 connections of interest as the graph edges. There were 21 (=7 choose 2) possible connections between seven regions, although only 13 of the CBGT circuit connections were considered. The nodes were ROIs in the CBGT circuit, as described previously. The fiber densities of connections between ROIs were selected as edge values. Fiber density considers the number of fibers connecting the regions and normalizes these edges based on the brain region area^53^. For each edge, fiber density was calculated as the product of connection density (CD) and connection efficacy (CE). The CD between two regions was defined as follows: 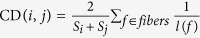, where *f* is the fiber connection between regions *i* and *j, l(f*) is the length of the fiber connection, and *S*_*i*_ is the surface area of region *i*. The CE was defined as the mean fractional anisotropy (FA) value along all fibers that connected the two regions. FA values are known to reflect axonal myelination and axonal diameter, which are important characteristics of neuronal fibers^53^,^54^. The edge values were entered into a matrix, whose elements were fiber density values. We adopted a simple network model that considered un-directed and weighted edges and was referred to as the connectivity matrix in this study.

### Statistical analysis

Group-wise differences in SBR for SPECT and fiber density for DTI were assessed using non-parametric permutation tests^55^,^56^. For each parameter, 45 participants were randomly assigned to the NC group, and the remaining participants were assigned to the PD group. The process was repeated 10,000 times, yielding a null distribution that would be used for group-wise differences. Significant differences (either in terms of SBR or fiber density) between groups were assessed if group-wise differences resulted in a value outside the 95% interval of the null distribution (determined by two-tailed tests with p < 0.05, corrected)^56^. All statistical analyses were performed using MATLAB (Mathworks Inc., USA).

### Predicting MDS-UPDRS scores

**Figure 7:**
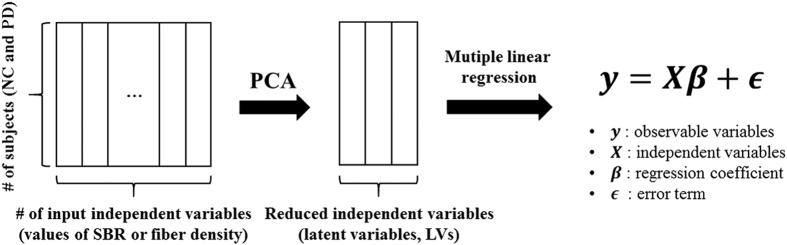
The conceptual diagram of PLSR. The PLSR is a combination of PCA and multiple linear regression. The PCA portion reduced the dimensionality of the data and thus removed the collinearity among independent variables. The dimensionality reduced data were used as independent variables to explore relationship between imaging features and the dependent variable (i.e., MDS-UPDRS score).

The SBR and fiber density values of identified regions and connections were used as independent variables in a PLSR framework to determine the relationships between the identified features and MDS-UPDRS score^57^,^58^,^59^. PLSR is effective when many independent variables are used to predict a dependent variable. PLSR is a combination of principal component analysis (PCA) and multiple linear regression^57^. The multi-collinearity issue among many independent variables could be avoided in the PCA part of PLSR^57^. A conceptual diagram of PLSR is given in Fig. 7. An important parameter in applying PLSR is determining the number of LVs. LV is a set of extracted components from the independent variables and having many LVs will lead to overfitting problems. The number of LVs was determined from the predicted residual estimated sum of squares (PRESS), which prevents overfitting the data. The number of LVs was increased until the value of PRESS no longer improved^57^. LOOCV was applied by assigning 89 (=90–1) subjects to the training set and the remaining subject to the test set. The number of LVs for each training set could theoretically change. Similar to number of LVs, the regression coefficients (β) could change for each training set. The ability of the SBR and fiber density values of identified regions to explain the MDS-UPDRS scores were quantified using explained variance statistics. The quality of prediction was assessed with p- and r-values between actual and predicted MDS-UPDRS scores. The RMS error value was computed to quantify differences between the predicted MDS-UPDRS and actual MDS-UPDRS scores^60^,^61^,^62^.

## Additional Information

**How to cite this article**: Son, S.-J. *et al*. Imaging analysis of Parkinson’s disease patients using SPECT and tractography. *Sci. Rep.* **6**, 38070; doi: 10.1038/srep38070 (2016).

**Publisher's note:** Springer Nature remains neutral with regard to jurisdictional claims in published maps and institutional affiliations.

## Supplementary Material

Supplementary Information

## Figures and Tables

**Table 1:** SBR values of ROIs with significant group-wise differences.

Region of interest	Specific binding ratio		*p*-value, corrected
	NC	PD	
Limbic cortex	1.1998 (0.0841)	1.2056 (0.0848)	0.361
*Associative cortex*	*0.9403 (0.0812*)	*0.8931 (0.0817*)	*0.003*
Sensorimotor cortex	0.9853 (0.0963)	0.9806 (0.0948)	0.427
Thalamus	1.7313 (0.1714)	1.6960 (0.1523)	0.173
Caudate	3.2831 (0.8017)	3.2491 (0.5110)	0.397
*Putamen*	*2.9202 (0.4583*)	*2.1156 (0.2506*)	<*0.001*
*Globus pallidus*	*1.8563(0.3975*)	*1.6241 (0.2254*)	<*0.001*

Mean value followed by standard deviations value in the parentheses were reported. Shaded rows denote regions which showed significant group-wise differences using permutation test.

**Table 2:** Fiber density values of connections with significant group-wise differences.

Connection	Fiber density		*p*-value, corrected
	NC	PD	
*Associative cortex – thalamus*	*7.72* × *10^−4^(4.92* × *10^−4^*)	*0.001 (6.66* × *10^−4^*)	*0.001*
Limbic cortex – caudate	0.002 (0.002)	7.86 × 10^−4^ (0.001)	<0.001
*Limbic cortex – putamen*	*0.003 (0.001*)	*0.002 (0.001*)	<*0.001*
Limbic cortex – thalamus	0.002 (7.52 × 10−4)	0.001 (8.51 × 10^−4^)	0.003
*Globus pallidus - putamen*	*0.002 (9.60* × *10^−4^*)	*0.001 (5.82* × *10^−4^*)	*0.002*
*Globus pallidus - thalamus*	*7.51* × *10^−4^ (4.82* × *10^−4^*)	*5.34* × *10^−4^ (4.63* × *10^−4^*)	*0.032*

Mean value followed by standard deviations value in the parentheses were reported. Fiber density is normalized fiber count per unit surface. Shaded rows denote connections which showed significant group-wise differences using permutation test and connections that contained identified regions of SBR analysis of Table 1 (i.e., associative cortex, putamen, and globus pallidus).

**Table 3:** Demographic data of the NC and PD groups.

	NC (n = 45)	PD (n = 45)	*p*-value
Sex (M:F)	21:19	25:15	0.39
Age	61.11 (10.67)	61.36 (10.13)	0.91
MDS-UPDRS score	0.51 (1.04)	20.51 (8.35)	<0.001

Values are reported as mean (standard deviation).

NC, Normal controls; PD, Parkinson’s disease patients; M, Male; F, Female; UPDRS, Unified Parkinson’s Disease Rating Scale.
