# Supplementary material for: Imaging analysis of Parkinson’s disease patients using SPECT and tractography
Source: Sci Rep. 2016 Nov 30;6:38070. doi: 10.1038/srep38070 (PMC5128922; doi:10.1038/srep38070)
Supplement: Supplementary Information [file srep38070-s1.doc]

**SUPPLEMENTARY INFORMATION**

**Imaging analysis of Parkinson’s disease patients using SPECT and tractography**

Seong-Jin Son, Mansu Kim, and Hyunjin Park

**METHOD**

**Validity of the prediction between actual and predicted MDS-UPDRS scores**
We predicted MDS-UPDRS scores using imaging features that could distinguish between NC and PD groups well. Both NC and PD groups were used to generate the independent variables to predict MDS-UPDRS score. There were unusually high of number cases with MDS-UPDRS score zero coming from the NC group. Dividing the MDS-UPDRS score into four intervals (0~9, 10~19, 20~29, and 30+), there were 48 cases (45 NC and 3 PD) in the range of 0~9, 22 PD cases in the range of 10~19, 12 PD cases in the range of 20~29 scores, and 8 PD cases in the range 30+. The mean number of cases in three intervals (10~19, 20~29, and 30+) was 14. We removed 34 NC cases from the first interval (0~9), which leads to having 14 cases (11 NC and 3 PD), to have evenly distributed number of cases covering all four intervals and re-performed the prediction analysis. The correlation between actual and predicted MDS-UPDRS scores was still significant (r = 0.5949, *p* < 0.001) compared to the results using all NC cases (*r* = 0.6854, *p* < 0.001).

**Figure**


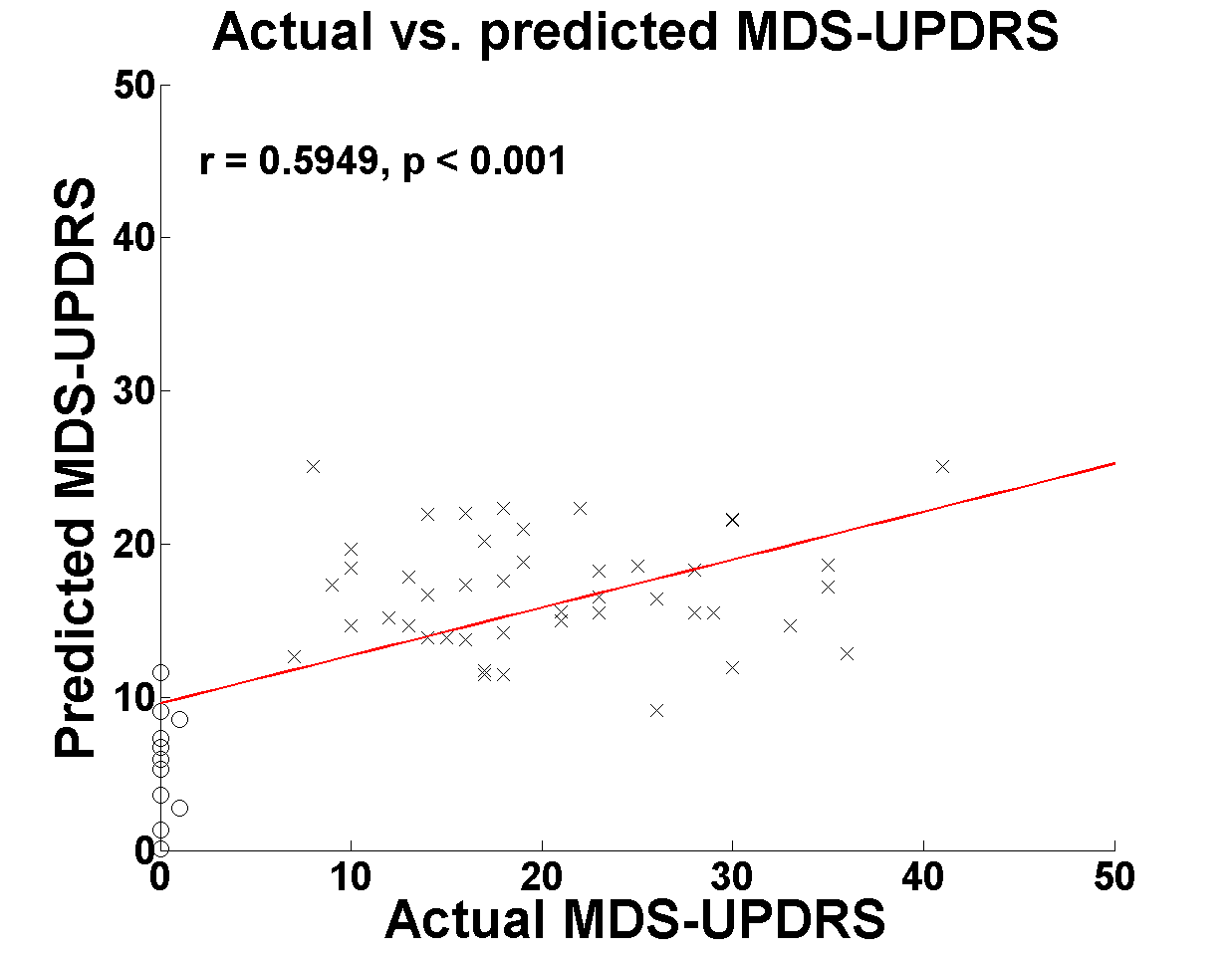


**Supplementary Figure S1.** Comparison of actual and predicted MDS-UPDRS scores between PD and NC. 34 NC cases with MDS-UPDRS scores in the interval (0~9) were removed to achieve evenly distributed number of cases covering full range of MDS-UPDRS scores. The correlation between actual and predicted MDS-UPDRS scores was still significant.

**TABLE**

**Supplementary Table S1. Most frequently used regression coefficients (β) for the PLSR model.** To predict the MDS-UPDRS scores, LOOCV procedure was performed 90 times (= number of total subjects) using identified regions and/or connections of both SPECT and DTI (rows 2-8), only DTI (rows 9-12), and only SPECT (rows 13-15).

| **Used independent variables** | **Region/Connection** | **Regression coefficient (**β**)** |
| --- | --- | --- |
| **SPECT & DTI** | Associative cortex | 0.13 |
|  | Putamen | -0.39 |
|  | Globus pallidus | -0.17 |
|  | Associative cortex – thalamus | 0.12 |
|  | Limbic cortex – putamen | -0.22 |
|  | Globus pallidus - putamen | -0.16 |
|  | Globus pallidus - thalamus | -0.16 |
| **DTI** | Associative cortex – thalamus | 0.19 |
|  | Limbic cortex – putamen | -0.35 |
|  | Globus pallidus - putamen | -0.25 |
|  | Globus pallidus - thalamus | -0.21 |
| **SPECT** | Associative cortex | 0.15 |
|  | Putamen | -0.44 |
|  | Globus pallidus | -0.19 |
